# Letter from Dr. Mercer

**Published:** 1846-10

**Authors:** 


### EDITORIAL DEPARTMENT.

*Letter from Dr. Mercer.*—We subjoin the Letter from Dr. Mercer alluded to in our last number. As almost every one is gratified with familiar descriptions by an eye-witness, of persons and places prominently associated with events or pursuits in which the reader is particularly interested, the accounts which Dr. M. gives of the hospitals and medical men of London will doubtless be acceptable. We hope our young correspondent will recollect his promise, and favor us with other letters.

LONDON, July, 1846.

DR. FLINT,

Dear Sir,—Agreably to Prof. Hamilton's request and my partial promise, I am seated to indite a letter for your Journal. But of what shall I write? The lions and great sights of London your readers are already as familiar with, as description can make them, and there is but little use in writing of diseases, since these are to be seen in every day life in America. I will endeavor to give some account of the hospitals and other things relating to medical matters here, which may perhaps possess some interest.

I presented my letters of introduction, was very kindly received on every side, and every facility was offered me to make acquaintance with the gentlemen connected with the different hospitals, and to visit their wards, of which I availed myself to the extent of visting some six or seven of their largest institutions.

St. Bartholomews' is one of the largest, oldest and richest of the London hospitals, and is situated but a few steps from the well known Old Bailey, of Newgate-St. It has been established for many centuries, and has been rebuilt time and again. At present it consists of four stately edifices, in the form of a hollow square. There are, however, many other buildings upon the hospital grounds, which are appropriated to various uses. The square, the courts, and yards are as destitute of vegetation as the surface of the Atlantic. In one of the main buildings, (which is chiefly devoted to offices and the residences of officers) upon the walls of the spacious staircase, are suspended two paintings, by Hogarth; one representing groups of diseased persons, coming to the hospital supported by their friends; the other the good Samaritan administering to their necessities. The lecture rooms, museum and library are in outer range of buildings. Our lecture rooms will lose nothing by comparison with those which I have seen here, in size, elegance or convenience. The seats have no backs, save here and there an iron post surrounded by a thick iron plate, some three or four inches wide. I used to think seats with good backs were sufficiently hard before night came, but these must be far less comfortable.

The museum is rich in both healthy and morbid specimens, but is not so large as might be expected, considering the time it has been accumulating.

The library is open every day to students, as a study or reading room, and contains about 4000 volumes. America has her representatives among them. Each student is allowed to have three books at his room at one time. All the more prominent medical periodicals are to be found upon the tables. Here are the busts of Harvey, Hunter, Abernethy, and other venerated men who made this the field of their teachings and professional labors..

At this, as at all other hospitals in London, patients are admitted free. A recommendation is required at some institutions. All however are open at all hours to cases of accidents.

St. B. has an annual income of not far from $235,000. A considerable portion of the principal is invested in real estate. This vast amount of property has been accumulating by the gifts of the benevolent from the time of its establishment. With such large resources they are abundantly able to keep up a fine and useful institution. Perhaps every thing is in better style here than at other hospitals, although some of the others are very richly endowed; and in passing through them so little difference is observable, that one can hardly give a decided preference to any.

St. B. supports 530 beds, 355 being allotted to surgical, and 175 to medical cases. The London Hospital, with an income of not far from $75,000, supports 320 beds, at which rate St. B. would have a considerable surplus, if the funds are used with the same economy as at the London Hospital. The medical and surgical officers here receive a small salary for their services out of the funds of the hospital, while at the London, and at some of the other hospitals, they get nothing, except what they receive from the students, which American students might think quite sufficient if they were to pay 30 dollars for a Prof. ticket, as that is not far from what they average here.

We visited the surgical wards with Mr. Lawrence, the author of a valuable work on the eye, &c. &c. He is not from 50 years of age, a shrewd, close observing man, with a bright, sparkling eye, and quite humorous in conversing with his patients. He is a popular lecturer, but not quite so popular as usual just now with the physicians, because in a recent address, he has claimed more honor due to the surgeon than to the physician. There were but few students following him through the wards, as there are few students in London at this season of the year. There are but few lectures delivered in the summer, and some of them have already closed. He had no cases of marked interest. In the venereal ward, which you generally find in the attic, was a young man with a syphilitic ulcer on the extremity of one his thumbs, which, Mr. L. remarked, was rather an unusual occurrence. Several presented themselves for admission, among others was a man with what was termed an adipose pterygium growing from both angles of the eye. He was not admitted.

I visited the medical wards with Dr. Roupell. He was very kind in showing me the hospital. He occnpies the chair of Materia

Medica. The diseases in his wards, at this season, were chiefly of a chronic character. Chronic rheumatism, granulated kidneys, chlorosis and its accompaniments, with a variety of nervous affections, comprise most of the disorders in his wards. In rheumatism the rule appears to be, if one medicine does not answer, try some other. They use no mercury in Bright's disease. An hysterical patient, whose bowels had been torpid for two or three days, the Doct. thought "should have them cleared out well with calomel and jalapa." The young gent. who had charge of prescriptions asked if he should give her calo. ii gr. The Doct. then ordered ℞, calo. iii gr., jal. xii gr. Just as we were leaving the wards, a young girl was brought in, aged about 16, in a state of insensibility. A well marked case of hysteria, "a good subject," said the Doct. with a wink, "for animal magnetism." The magnetism that he thought proper to use, was a magnetic current of cold water, poured in a small stream from a considerable height upon her head—he thought that would bring her to consciousness—to be followed with the usual remedies. Over each patient's bed is to be seen the name of the physician, or surgeon, who treats them; also upon a printed form the patient's name, age, occupation, day of admission, name of disease, some of the more prominent symptons, and the medicine that he has taken from day to day. A more detailed history is kept by the physician or surgeon's pupils, and dressers, who, in a measure, have charge of patients in their absence, so as to give a correct history of the changes from time to time, as they do not visit their patients every day. A great number were taking their pint of porter per day, some less, others had a good allowance of wine. The rule seems to be to give all patients porter or wine, unless there are symptoms which directly contraindicate their use. Patients were taking more porter and wine here than at any other hospital. This may be owing to the cases being chiefly those of chronic forms of disease. When they have as many temperance men in England as we have in America, they may dispense in a measure with its use. But the Lord only knows when that will be, not in this generation, unless a mighty change is effected.

We heard a lecture on botany, from Doct. Fane, which was about the same as other men's lectures. One thing about him seemed strange enough,—he lectured enrobed in a richly-trimmed silk gown. The students in attendance did not exceed thirty. At the Middlesex hospital we listened to another lecture on the same subject. This was on Saturday, and there were but two students present. Whether the students were absent, or these two were all that attended I know not.

The management of the several hospitals is very much the same, and a general account of one answers very well for them all. There is one thing at St. B. that is different, or something beyond what they have at any other medical school here. It it what they term a Collegiate Institute, but will be better understood by calling it a boarding house. The buildings are situated within the hospital walls, and will accomodate about 30 students. They are under the same general regulations as regards study and moral dicipline, as at literary colleges. The rooms are very commodious, and every thing about them as it should be. They have breakfast and tea served in the rooms; but dinner in a common hall, at 5 P. M. For all this the students pay from thirty to thirty-five dollars per month, according to the rooms and the attendance they have.

Mr. S. Cooper gave me a line to Mr. Arnott, at the Middlesex hospital. I saw him, but it was not his day for visiting the wards; but with an introduction to Mr. Shaw, I visited them with him. They have a large number of scrofulous children, some of them very bad cases. They are using the cod-liver oil as a common remedy in the disease, and think it answers better than anything else they have used. One child, at three or four years of age, was taking ʒi three times per day. There were three or four fractured patellas, which they were treating by position alone. A case of punctured femoral artery and vein, by a spike. It was not clear at the time of the injury, whether the artery was punctured or not. It was thought not, and was treated by compression. Now, a peculiar thrill can be heard and felt, supposed to be the blood rushing from the artery into the vein. Patient is doing well. There is a ward in this hospital devoted entirely to scirrhous diseases. Some person, unknown for many years,—and still unknown to me—left a sum of money to establish and maintain this charity, in which there now from six to eight patients. Back of the hospital buildings there is a small garden. It must be gratifying to patients who are tortured in body, and depressed in mind, to view one green spot of earth, be it ever so small, amid this wilderness of buildings.

*St. George's Hospital.*—The buildings are of a modern order of architecture, and have a lofty and commanding appearance. They cannot have been erected long. It is situated opposite Hyde Park Corner, and nearly across the street from the residence of the Duke of Wellington, in the midst of the wealth and aristocracy of England. The hospital servants, in a measure, keep pace with those of the gentry around them, the porter at the door being dressed in livery. I visited here on Thursday, their operating day, with the expectation of seeing an ovarian tumour removed; but the friends of the patient were so strenuous in their opposition, that the operation was not performed. The case seemed to excite considerable interest, and even in London would have been rather a rare operation. There is said to be great sympathy between the reproductive and the nutritive organs. It might have been so in this case, for though we saw not an operation for ovarian disease, we did for mammary. A small fibrous tumour was dissected out. The operator's name I have forgotten. He remarked that it was not malignant, but might grow as large as a child's head. It should be removed when small, and there would be no farther trouble. Their operating theatre has not its superior for light, as the roof is all skylight. A post-mortem, in the dead house, followed the operation, of a man who had suddenly died, complaining mostly of dyspnœa, and of his left arm and hand being cold and numb. On dissection, there was found a large aneurism of the aorta, which had burst within the cavity of the pericardium. It was completely filled with coagulated blood, which was taken out by the double-handful. Sir B. Brodie lectures at this school on special points in surgery, and Doct. R. Lee on midwifery.

*University College Hospital.*—The faculty here presents a good array of talent. Docts. Williams, A. T. Thomson and Walshe, and Messrs. S. Cooper, Liston and Quain, with Doct. Graham in the Chair of Chemistry. We had the pleasure of visiting Mr. Liston's wards with him, though we saw and learned less than at some other places. There were more than double the number following him than we had seen following any other gentleman, so that there was far too many for all to see and hear. There was a native of some part of the East Indies, in the crowd of students. His features were regular and well-formed, and he was by no means a bad looking man, though his complexion is a shade or two darker than our Indians. To judge from the dress and general appearance of the students here, they would seem to be of rather a higher grade in society than at some other places, and the higher fees would denote the same. Mr. Liston was driven to the hospital in his carriage, with livery servants before and behind, which style is generally supported by the profession here. He is a large and rather coarse featured man. His surgery for the day consisted mostly in the removal of a soft polypus from the nose by forceps. The patient said it had been removed two or three times before, once with a wire, which was derided. While he was twisting it from its attachments, the patient rose from his seat, when Mr. L. cried out, sit d-o-w-n, sit d-o-w-n, and the more he twisted and pulled, and said sit down, the more the patient stood up. After it was removed the patient blew some blood on him from his nose; "don't blow your nose in my face!" said he, and, at the same time, pushed his head down into the basin he was holding; "now blow away as much as you like." He passed his little finger far back in the nares for more, which could be readily done on the patient as on himself, judging from the visible size of the olfactory organs in both. There was a child presented whose shoulder joints had been much injured by being frequently lifted by her arms. The ligaments were badly stretched, and the bones deformed.

